# Maintained Clinical Remission in Ankylosing Spondylitis Patients Switched from Reference Infliximab to Its Biosimilar: An 18-Month Comparative Open-Label Study

**DOI:** 10.3390/jcm8070956

**Published:** 2019-07-02

**Authors:** Evripidis Kaltsonoudis, Eleftherios Pelechas, Paraskevi V. Voulgari, Alexandros A. Drosos

**Affiliations:** Rheumatology Clinic, Department of Internal Medicine, Medical School, University of Ioannina, 45110 Ioannina, Greece

**Keywords:** ankylosing spondylitis, infliximab, biosimilar, switching

## Abstract

Background: Switching from reference infliximab (RI) to biosimilar infliximab (BI) had no detrimental effects on efficacy and safety. However, long-term follow-up data is missing. Objective: To evaluate patients with Ankylosing Spondylitis (AS) in clinical remission who were switching from RI to BI, in terms of the safety and efficacy of this, in a long-term fashion. Methods: One hundred and nine consecutive unselected AS patients were investigated. All were naïve to other biologics and were followed-up at predefined times receiving RI. Patients in clinical remission were asked to switch from RI to BI. Those who switched to BI were compared with a matched control-group receiving continuous RI. During follow-up, several parameters were recorded for at least 18 months. Disease activity was measured using the Bath Ankylosing Spondylitis disease activity index (BASDAI), and the Ankylosing Spondylitis disease activity score (ASDAS), using the C-reactive protein. Remission was defined as BASDAI < 4 and ASDAS < 1.3. Results: Eighty-eight patients were evaluated (21 excluded for different reasons). From those, 45 switched to BI, while 43 continued receiving RI. No differences between groups regarding demographic, clinical and laboratory parameters were observed. All patients were in clinical remission. During follow-up, five patients from the BI-group and three from the maintenance-group discontinued the study (4 patients nocebo effect, 1 loss of efficacy). After 18 months of treatment, all patients in both groups remained in clinical remission. No significant adverse events were noted between groups. Conclusion: BI is equivalent to RI in maintaining AS in clinical remission for at least 18 months.

## 1. Introduction

CTP-13 (Inflectra®, Remsima®) the biosimilar infliximab (BI) has been granted all indications, including Ankylosing Spondylitis (AS), of the reference infliximab (RI) in several countries [1]. Clinical evidence for the approval of BI has been obtained from pivotal studies on patients with AS (PLANETAS) [2] and rheumatoid arthritis (RA) (PLANETRA) [3]. Switching from RI to its biosimilar had no detrimental effects on efficacy, safety or immunogenicity compared with continuous RI therapy [4,5]. Therefore, BI is an efficacious alternative to RI in patients with inflammatory arthritis [6]. On the other hand, there are reports emphasizing the role of shared-decision making with patients when it comes to switching to a biosimilar product in order to achieve a better acceptance and higher retention rate, minimizing the nocebo effect [5]. However, long-term follow-up data is missing. The aim of our study was to investigate if BI is equivalent to RI to maintain patients with AS in clinical remission compared with continuing RI in a long-term fashion. 

## 2. Materials and Methods

This is a single-center prospective observational cohort study with a total number of 109 consecutive unselected patients with AS who were treated with RI in a tertiary university center. All patients were followed-up at predefined times receiving RI (5 mg/kg/8 weeks) intravenously and were naïve to previous biologic treatments. Patients who were in clinical remission were asked to switch from RI to BI using the same therapeutic dose after shared-decision making. The allocation of the patients was done randomly using an internet-based allocation program in order to minimize any selection bias (Random.org). Patients switched to BI were compared with a matched control group receiving continuous RI. The switching period was from January 2017 until June 2017 and patients were followed-up until December 2018. During follow-up, the demographic, clinical, and laboratory parameters as well as comorbidities were all recorded for at least 18 months. In addition, all adverse events as well as serious adverse events according to the Food and Drug Administration (FDA.gov) were also recorded. Disease activity was measured using the Bath Ankylosing Spondylitis Activity Index (BASDAI) [7] and the Ankylosing Spondylitis Activity Score (ASDAS) [8,9] using the C-reactive protein (CRP). Clinical remission was defined if patients had BASDAI < 4 and ASDAS < 1.3. Statistical analysis was performed using SPSS statistics version 20.0 (IBM Corporation, Armonk, NY, USA) We used the paired samples t-test for variables with normal distribution and Wilcoxon signed ranks test for variables which were not normally distributed. A *p*-value < 0.05 was considered statistically significant. Written informed consent was obtained from all patients, and the study has been approved by the clinical Research Ethic Committee of the University Hospital of Ioannina, according to the principles in the Declaration of Helsinki (197/2-12-2016).

## 3. Results

**Figure 1:**
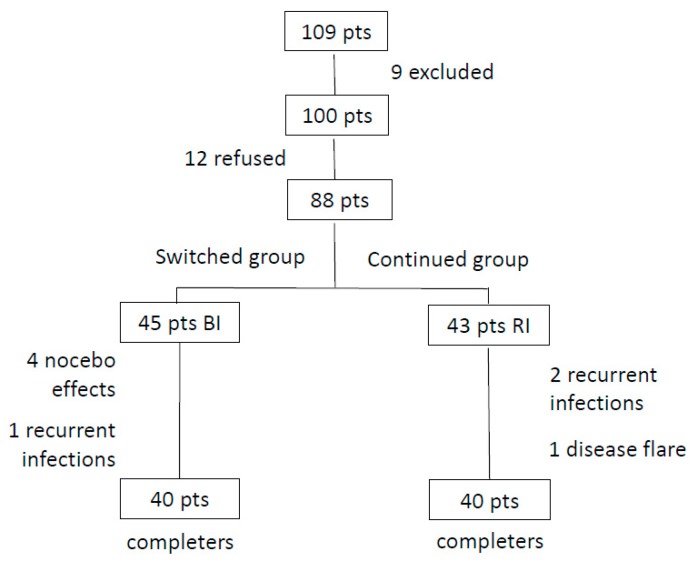
Flow chart of AS patients treated with infliximab.

Twenty-one patients were excluded: 9 because they were not in clinical remission and 12 refused to switch from RI to its biosimilar. Thus, the final results comprise 88 patients. From these patients, 45 switched to BI, while 43 continued receiving RI (Figure 1). The demographic and clinical characteristics of our patients are depicted in Table 1. There were no differences between groups regarding the demographic, clinical and laboratory parameters. All patients were in clinical remission with low BASDAI and low ASDAS for approximately 3 years. During the follow-up period, 5 patients from the switched group and 3 from the continuing group discontinued the study (Figure 1). Four patients receiving BI presented nocebo effects after the second infusion while one had recurrent urinary tract infections. The patients with nocebo effects experienced nonspecific, subjective complaints such as headache, somnolence, dizziness, arthralgias, fatigue and pain. The clinical examination of these patients was unremarkable, and the acute phase reactants were within normal limits. These patients were switched to RI. Three responded well, while the fourth did not, and was changed to interleukin-17 (IL-17) inhibitor with good results. On the other hand, from the patients who continued receiving the RI, two patients presented recurrent upper respiratory tract infections while one had a disease flare-up. These patients were treated with an IL-17 inhibitor and responded very well. After 18 months of follow-up, all patients in both groups remained in clinical remission with low BASDAI, low ASDAS as well as low erythrocyte sedimentation rate (ESR) and CRP (Table 2). No significant adverse events, serious adverse events or any comorbidities were noted between the studied groups (Table 3).

## 4. Discussion

Biosimilars represent an important new generation of drugs in a rheumatologist’s armamentarium [10]. Biosimilars have been approved by the European Medical Association (EMA) for rheumatologic indications and those for which the biological originator is no longer patent-protected. CTP-13, under the commercial name Inflectra/Remsima, was the first biosimilar approved by the EMA in 2013 [1]. Approval of BI was based on findings from two pivotal trials in AS [2] and RA [3]. Data from open-label extension studies of the original trials for AS have been reported [5]. Current data supports the proposal that it was possible to switch from RI to BI without any detrimental effects on safety and efficacy [5]. In addition, all available data regarding switching from RI to its biosimilar are reassuring. Switching is also recommended in the European League Against Rheumatism (EULAR) guidelines [11]. Indeed, a 52-week double-blind trial supports the efficacy and safety of the switch from RI to its biosimilar in patients with stable disease [12]. However, long-term follow-up data are required to confirm the efficacy and safety of the switch. The present study tries to cover this gap.

In our study, 88 AS patients receiving RI who were in clinical remission were asked to switch to BI. Half of them received BI, while the rest continued receiving RI. After 18 months of follow-up, no differences of clinical efficacy and safety were found between groups. Both groups remained in clinical remission. Our findings are in line with the PLANETAS study despite the fact that they used different tools in assessing disease activity [2]. Five patients from the BI group and three from the RI group discontinued the treatment. In the switched group, four patients discontinued the treatment due to nocebo effects. Nocebo effects are complex and individualized clinical phenomena that can induce new worsening pain, nonspecific subjective complaints such as malaise, fatigue, headache, weakness and others which are mainly induced by the patients’ negative expectations [13]. Thus, physicians should be aware of the potential appearance of nocebo effects which may hinder the transition to biosimilars in some patients [14]. Our patients responded very well to switching from RI to its biosimilar. The reason for this could be the clinical state of the patients that are in clinical remission. Our results are in line with those of other investigators who reported a high retention rate of switching to biosimilars if the patients are stable [5,12]. Another reason could be that the switching was after discussion and decision-making with the patients. Evidence-based recommendations are available for several conditions in order to guide physicians in the switching process with biologics. Data suggests that shared-decision making leads to a better therapeutic response with fewer nocebo effects in contrast to non-medical switching [15,16]. The limitation of our study is that we included a small number of patients. On the other hand, the strength of our study is that it is the longest comparative study regarding switching from the RI to BI in AS.

When biosimilars appeared in the market, they not only had a lower price but also led to the price erosion of the reference products. In our study, there were no differences between the studied groups, and despite the fact that we did not make a cost-effectiveness analysis, we assume that the cost of the BI per patient is lower than that of the RI. Our study offers the promise of substantial savings relative to the RI product, enabling more AS patients to access biological therapy and reducing the cost associated with expensive biological treatment [17]. 

## 5. Conclusions

This is the first study in which AS patients in clinical remission receiving RI who were switched to BI remained in clinical remission for at least 18 months. We demonstrated that BI is equivalent to RI in maintaining AS patients in clinical remission.

## Figures and Tables

**Table 1:** Demographic and clinical characteristic of Ankylosing Spondylitis (AS) patients at switching.

Parameters	Switched Group (BI) *n*:45	Continued Group (RI) *n*:43	*p*-Value
Mean age (years) (SD)	36.1 (4.6)	35.7 (4.3)	NS
Male/female	39/6	40 (3)	NS
Mean disease duration (years) (SD)	7.8 (3.0)	7.6 (2.8)	NS
Mean follow-up (years) (SD)	7.0 (1.1)	6.9 (0.9)	NS
BMI (kgr/m^2^) >25	5 (11.1)	4 (9.3)	NS
Current smokers n (%)	10 (22.2)	8 (18.6)	NS
Ex-smokers n (%)	9 (20)	9 (20.9)	NS
Mean treatment with BI/RI	6.4 (0.9)	6.5 (0.8)	NS
Mean treatment with RI and clinical remission (years) (SD)		3.6 (0.8)	NS
Axial disease n (%)	45 (100)	43 (100)	NS
Peripheral disease n (%)	4 (9)	3 (7)	NS
Methotrexate intake n (%)	3 (7)	2 (5)	NS
Mean BASDAI (SD)	3.7 (0.2)	3.6 (0.4)	NS
Mean ASDAS (SD)	1.0 (0.2)	1.1 (0.2)	NS
Mean ESR mm/h (SD)	18.5 (2.2)	19.3 (1.7)	NS
Mean CRP mg/L (SD)	6.0 (0.8)	5.8 (0.6)	NS

BI, biosimilar infliximab; RI, reference infliximab; SD, standard deviation; BMI, body mass index; BASDAI, bath ankylosing spondylitis activity index; ASDAS, ankylosing spondylitis disease activity score; ESR, erythrocyte sedimentation rate; CRP, C-reactive protein; NS, non-statistical.

**Table 2:** Response to treatment in AS patients switched to BI versus those continuing RI.

Parameters	Switched Group (BI)	Continued Group (RI)	*p*-Value
**At switching**			
BASDAI (SD)	3.7 (0.2)	3.6 (0.4)	NS
ASDAS (SD)	1.0 (0.2)	1.1 (0.2)	NS
ESR mm/h (SD)	18.5 (2.2)	19.3 (1.7)	NS
CRP mg/l (SD)	6.0 (0.8)	5.8 (0.6)	NS
**End of the study**			
BASDAI (SD)	3.7 (0.4)	3.8 (0.2)	N5
ASDAS (SD)	1.0 (0.2)	1.1 (0.1)	NS
ESR mm/h (SD)	19.5 (1.5)	20.0 (1.6)	NS
CRP mg/l (SD)	6.0 (1.0)	6.1 (1.1)	NS

BI, biosimilar infliximab; RI, reference infliximab; SD, standard deviation; BASDAI, bath ankylosing spondylitis activity index; ASDAS, ankylosing spondylitis disease activity score; ESR, erythrocyte sedimentation rate; CRP, C-reactive protein; NS, non-statistical.

**Table 3:** Adverse events during follow-up in AS patients switched to BI versus those continuing RI.

Adverse Events * *n* (%)	Switched Group (BI)	Continued Group (RI)	*p*-Value
Upper respiratory tract infections	3 (6.6)	2 (4.6)	NS
Urinary tract infections	2 (4.4)	2 (4.6)	NS
Skin infections	2 (2.2)	1 (2.3)	NS
Increased liver enzymes	2 (4.4)	2 (4.6)	NS
Diarrhea	1 (2.2)	2 (4.6)	NS
Viral infections	2 (4.4)	1 (2.3)	NS
Headache	1 (2.2)	0 (0)	NS
Hypertension	1 (2.2)	1 (2.3)	NS

*, not requiring discontinuation; BI, Biosimilar Infliximab; RI, Reference Infliximab; n, number of patients; NS, Non-statistical.
